# Intra-articular temperatures of the knee in sports – An in-vivo study of jogging and alpine skiing

**DOI:** 10.1186/1471-2474-9-46

**Published:** 2008-04-11

**Authors:** Christoph Becher, Jan Springer, Sven Feil, Guiliano Cerulli, Hans H Paessler

**Affiliations:** 1 Department of Orthopaedic Surgery, Phillips University, Marburg, Germany; 2 Center for Knee & Foot Surgery/Sports Trauma, ATOS Clinic, Heidelberg, Germany; 3 "Lets people move" Institute, University of Perugia, Perugia, Italy

## Abstract

**Background:**

Up to date, no information exists about the intra-articular temperature changes of the knee related to activity and ambient temperature.

**Methods:**

In 6 healthy males, a probe for intra-articular measurement was inserted into the notch of the right knee. Each subject was jogging on a treadmill in a closed room at 19°C room temperature and skiing in a ski resort at -3°C outside temperature for 60 minutes. In both conditions, temperatures were measured every fifteen minutes intra-articulary and at the skin surface of the knee. A possible influence on joint function and laxity was evaluated before and after activity. Statistical analysis of intra-articular and skin temperatures was done using nonparametric Wilcoxon's sign rank sum test and Mann-Whitney's-U-Test.

**Results:**

Median intra-articular temperatures increased from 31.4°C before activity by 2.1°C, 4°C, 5.8°C and 6.1°C after 15, 30, 45 and 60 min of jogging (all p ≤ 0.05). Median intra-articular temperatures dropped from 32.2°C before activity by 0.5°C, 1.9°C, 3.6°C and 1.1°C after 15, 30, 45 and 60 min of skiing (all n.s.). After 60 minutes of skiing (jogging), the median intra-articular temperature was 19.6% (8.7%) higher than the skin surface temperature at the knee. Joint function and laxity appeared not to be different before and after activity within both groups.

**Conclusion:**

This study demonstrates different changes of intra-articular and skin temperatures during sports in jogging and alpine skiing and suggests that changes are related to activity and ambient temperature.

## Background

Intra-articular temperatures were well investigated in recent years. The first analysis of intra-articular temperatures in humans was published in 1949 by Horvath and Hollander based on the thought that arthritic joints were not only warm to the touch but had a higher intra-articular temperature as compared with unaffected joints [1]. A statistically significant decline in intraarticular knee temperature with the application of ice and compression to the skin was first documented by Martin et al. in 2001 [2]. Several authors have investigated the influence of application of cold, heat or pathological conditions such as synovitis on the change of the physiological intra-articular temperature of the knee [3-8]. Beneficial effects of cooling of joints after acute injury or in the postoperative period were reported with shorter hospitalization, less pain with fewer need for analgesics and less swelling [9-13]. Improved range of motion progression and exercise tolerance are other constituted advantages [9-14]. However, cold or heat to a joint is of concern to have negative influence on muscle performance and increased risk of injury and therefore discussed controversial [15-21].

To our best knowledge, no reports exist about the changes of the intra-articular temperatures and skin surface temperatures of the knee during sports activity. The objectives of the present study are to evaluate differences of intra-articular and skin surface temperatures related to activity and ambient temperature. Furthermore, a possible impact on joint function and laxity is examined.

## Methods

6 healthy males were included in the study after approval by the institutional review board and informed consent. The average age of the participants was 29.3 years (range: 27 – 32 years). The average Body Mass Index (BMI) was 23.61 (range: 22.78 – 25.25). On examination, all participants had full, pain-free range of motion, stable ligaments and no effusion or tenderness to palpation.

**Figure 1:**
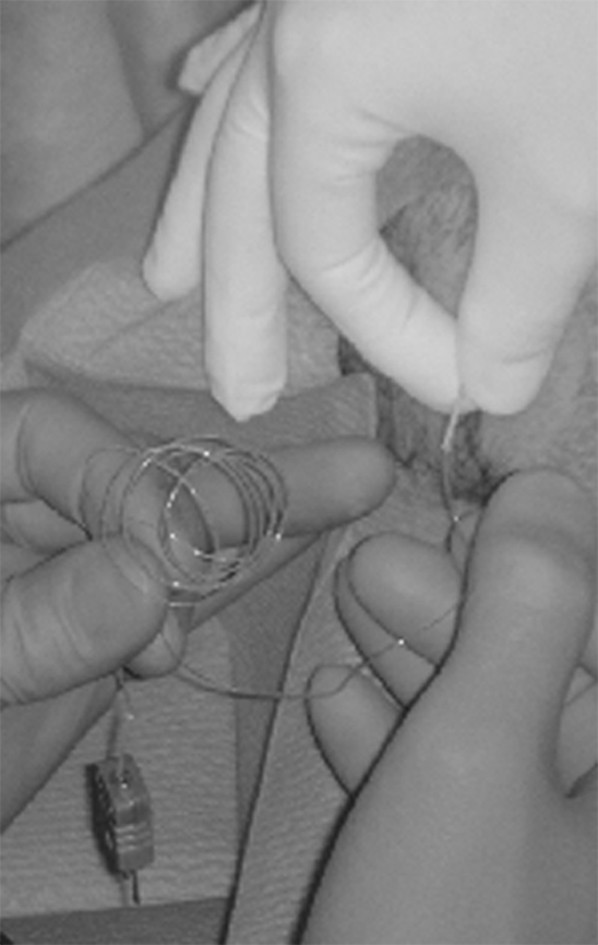
Intra-articular insertion of the probe into the notch.

For preparation of the implantation procedure, the subject was sitting with the knee flexed to 90°. The probe for the intra-articular measurement was inserted intra-articulary under local anaesthesia (Bupivacain 0.5%) into the notch under sterile conditions through an 18-gauge needle introduced to the medial aspect of the right knee (Fig. 1). All implantations were done by the senior investigator (HHP). The introduced device was a flexible Teflon coated thermocouple probe (Model IT-18, Physitemp Instruments Inc, New Jersey, U.S.A.; max. temp. 150°, time constant 0.1 sec., sensor lead 0.25" dia, length 3 feet). A second and third thermocouple probe (Model SST-1, Physitemp Instruments Inc, Clifton, New Jersey, U.S.A; 10 Kt. Gold sensor disc, not isolated, max. temp. 90°, time constant 0.15 sec.) was placed on the skin 2 cm proximal of the patella and fixed with adhesive tape. The temperature was recorded with the TH-8 Thermalert Monitoring Thermometer (Physitemp Instruments Inc, Clifton, New Jersey, U.S.A.; accuracy of 0.1°C ± 0.2%, stability ± 0.1°C). These devices were successfully used by other authors in the same application [8].

### Study protocol jogging

All subjects wore common sport shorts that ended above the knees for performing the run. Each participant was jogging on a treadmill at a speed of 6.5 km/h for 60 minutes. Every fifteen minutes, the temperature was measured intra-articulary and at the skin.

### Study protocol skiing

The implantation of the probe was done in a harborage next to the slope. Each participant used skiing suits of the same style and material (Shell: 100% Nylon; Trim: 100% Polyester; Lining: 100% Nylon) without skiing underwear. The slope was rated at an easy degree of difficulty with a length of 800 meter. Ascent was provided by a chair lift. The participants were requested to ride on a basic level to provide comparable activity levels. Every fifteen minutes, the temperature was measured intra-articulacy and at the skin. For that purpose, the skiing suit had to be released in a manner to set the probe free. Measurements were done in outside conditions next to the entrance of the chair lift and were performed to the exact point in time.

The outside temperature in the laboratory where the trial was done was 19°C; the outside temperature at the skiing resort was -3°C at the entrance of the chair lift. The outside temperatures did not change during the trial. Jogging and skiing was each started around 10 o'clock am. Between the jogging and skiing activity, the subjects had one day of rest for recovery.

Three different tests were used to evaluate joint function and laxity (neuromuscular function, intermuscular coordination, strength, proprioception and anterior-posterior stability). Every test was performed before and after the implantation of the probe as well as after the activity. The tests were performed in the same manner under equal conditions in the lab and in the harborage next to the slope. The mean value of three independent measurements was taken for data analysis. Every subject underwent three repetitions of every testing modality prior to the trial to get used to the tests. For evaluation of passive anterior knee joint stability, a Lachman-test was performed using the KT 2000 knee arthrometer (Medmetrics, San Diego, Calif., U.S.A). Strength and intermuscular coordination were investigated by a single leg hop test. This test was used by many researchers to study knee function in ACL-deficient persons particularly because hopping is more challenging than walking or jogging and is thought by some to more closely represent the demand of high level sports [22,23]. The joint position sense was evaluated using a passive angle reproduction test using a standard goniometer. Patient eyes were closed in order to eliminate visual stimuli.

For statistical analysis descriptive statistics were summarized with medians and ranges. Complete data sets were available for 2 × 6 persons. All data were tested for deviation from the normal distribution within the groups using Box-and-Whisker-Plots. Temperature changes over time and differences within one group were assessed using a paired Wilcoxon sign rank sum test. A Mann-Whitney-U-Test was used to assess differences between groups. These non-parametric two-tailed tests are not based on assumptions about the normal distribution. The Box-and-Whisker Plot displays the first and third quartiles as the ends of the box, the maximum and minimum as the whiskers and the median as a vertical bar in the interior of each box. Extreme values did not appear. All tests were two-sided and a p-value ≤ 0.05 was considered significant. Descriptive mean and 95%-confidence intervals were calculated for all variables of joint function. Data analysis was performed with SPSS for Windows 12.0 (SPSS inc. Chicago, Illinois, USA).

## Results

### Temperature measurements

**Figure 2:**
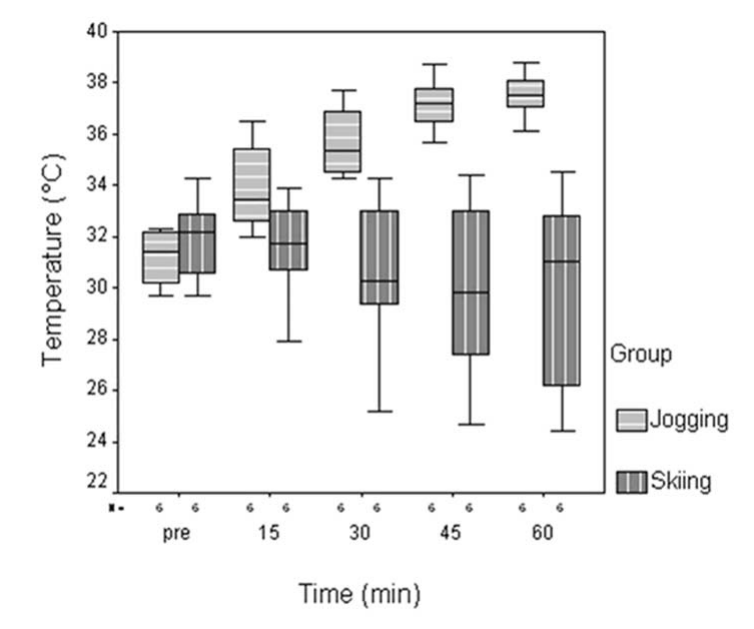
**Intra-articular temperatures measured before and during jogging and skiing activity over one hour**. Significant increase of intra-articular temperatures was measured after 15, 30, 45 and 60 min in the jogging group. No significant changes were observed in the skiing group.

The median initial intra-articular temperatures (Fig. 2) of the jogging group (31.4°C, min/max-range 29.7°C/34.3°C) and the skiing group (32.2°C, min/max-range 29.7°C/32.3°C) revealed no statistical significant difference (p = 0.377). Compared to the baseline temperature values, median intra-articular temperatures in the jogging group increased by 2.1°C, 4°C, 5.8°C and by 6.1°C after 15 min (33.5°C; min/max range 32°C/36.5°C; p = 0.05), 30 min (35.4°C; min/max range 34.3°C/37.7°C; p < 0.05), 45 min (37.2°C; min/max range 35.7°C/38.7°C; p < 0.05) and 60 min (37.5°C; min/max range 36.1°C/38.8°C; p < 0.05) of activity. Median intra-articular temperatures in the skiing group dropped by 0.5°C, 1.9°C, 3.6°C and 1.1°C after 15 min (31.7°C; min/max range 27.9°C/33.9°C; n.s.), 30 min (30.3°C; min/max range 25.2°C/34,3°C; n.s.), 45 min (29.8°C; min/max range 24,7°C/34,4°C ; n.s.) and 60 min (31.1°C; min/max range 24.4°C/34.5°C; n.s.) of activity.

**Figure 3:**
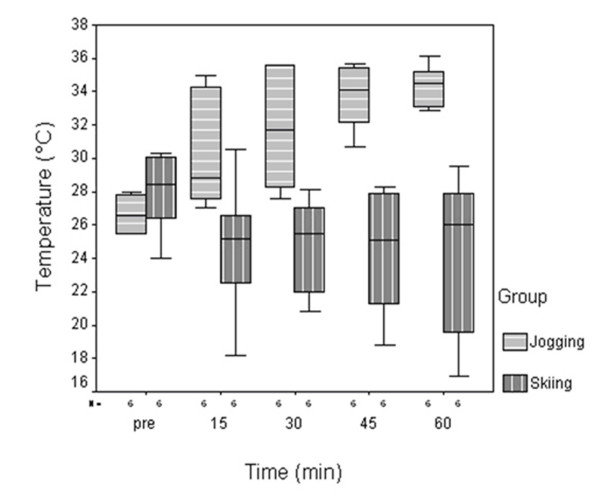
**Skin surface temperatures at the knee measured before and during jogging and skiing activity over one hour**. Significant increase of the skin surface temperatures was measured after 15, 30, 45 and 60 min in the jogging group. No significant changes were observed in the skiing group.

The skin surface temperatures at the knees (Fig. 3) revealed median increases in the jogging group from 26.6°C (min/max range 25.5°C/28.0°C) before activity by 2.3°C, 5.1°C, 7.6°C and 7.9°C at 15 min (28.9°C; min/max range 27.0°C/35.0°C ; p < 0.05), 30 min (31.7°C; min/max range 27.6°C/35.6°C ; p < 0.05), 45 min (34.2°C; min/max range 30.7°C/35.7°C; p < 0.05) and 60 min (34.5°C; min/max range 32.9°C/36.1°C; p < 0.05) of activity. The temperatures at the skiing group dropped from 28.5°C (min/max range 24.0°C/30.3°C) before activity by 3.3°C, 3.0°C, 3.4°C and 2.5°C after 15 min (25.2°C; min/max range 18.2°C/30.5°C; n.s.), 30 min (25.5°C; min/max range 20.8°C/28.1°C; n.s.), 45 min (25.1°C; min/max range 18.8°C/28.3°C; p = 0.05) and 60 min. (26.0°C; min/max range 16.9°C/29.5°C; n.s.) of activity.

Comparing the intra-articular with the skin knee temperature after 60 minutes of skiing (jogging), the median intra-articular temperature was 19.6% (8.7%) higher than the skin temperature at the knee.

### Joint function and laxity

The ability to perform the single leg hop test seemed not to be decreased after activity compared to the situation before activity in both groups. However, implantation of the probe appeared to decrease the ability for hopping (Table 1). Comparing the two groups for their ability for hopping before activity revealed no obvious differences. The skiing group, however, slightly improved their median ability for hopping after the activity, whereas the joggers could not achieve the same results as prior to the trial (Table 1).

**Table 1:** Single leg hop test (Median (cm) min/max-range)

Group	Pre_implantation	Post_implantation – pre_activity	Post_activity
Jogging	119.5 (109.7/150.3)	99.2 (91.2/107.4)	115.7 (102.7/157.3)
Skiing	124.5 (111.3/135.0)	99.2 (90.1/102.3)	130.0 (103.7/146.0)

Implantation of the probe (post_implantation – pre activity) appeared to decrease the ability to perform the single leg hop test

Joint laxity (Table 2) and joint position sense (Table 3) within the groups appeared not to be influenced by the implantation of the probe or the activity. Implantation of the probe, however, appeared to decrease accurate reproduction of joint angles.

**Table 2:** KT 2000 (Median (mm) ± min/max-range)

Group	Pre_implantation	Post_implantation – Pre_activity	Post_activity
Jogging	9.0 (7.0/10.0)	8.0 (7.0/10.0)	8.5 (7.0/10.0)
Skiing	9.5 (7.0/10.0)	9.5 (7.0/10.0)	10.0 (7.0/10.0)

Joint laxity within the groups appeared not to be influenced by the implantation of the probe or the activity

**Table 3:** Joint position sense (Mean difference between predetermined and reproduced angle ± SD)

	30°	60°	90°
	Pre_implantation		
Jogging	5.0° ± 2.89°	7.5° ± 3.8°	5.8° ± 4.5°
Skiing	5.0° ± 2.9°	6.7° ± 3.7°	4.2° ± 6.1°
			
	Post_implantation – Pre_activity		
Jogging	11.7° ± 6.9°	8.3° ± 3.7°	9.2° ± 7.3°
Skiing	7.5° ± 9.0°	6.67° ± 3.73°	8.83° ± 3.73°
			
	Post-activity		
Jogging	6.7° ± 7.5°	5.0° ± 4.1°	7.5° ± 2.5°
Skiing	3.3° ± 2.4°	5.8° ± 1.9°	7.5° ± 3.8°

Implantation of the probe (post_implantation – pre activity) appeared to decrease the joint position sense

## Discussion

Changes of intra-articular temperatures were well investigated in the field of orthopaedic surgery to study possible beneficial mechanisms of cold application and to evaluate potential negative effects on joint homeostasis [2,5,7,9-13,24-26]. However, up to date, we had no knowledge about the changes and possible effects of intra-articular temperature changes related to activity and ambient temperature.

Haimovici measured the intra-articular temperature of healthy human knees to be 32.8°C on average [3]. The initial temperatures in our study group were slightly lower but consistent with the findings of other investigators [7,8]. Although our healthy young male subjects performed knee demanding activity by alpine skiing, the median intra-articular and skin knee temperatures dropped over the testing time of 60 minutes. Thus, the cool outside temperature appeared to influence the intra-articular and skin knee temperatures. However, in contrast to studies with cooling, but without activity demand of the knee [8,24], the intra-articular temperatures did not drop significantly.

In the jogging group, median intra-articular temperatures increased to 37.5°C after 60 min of activity, which was 8.7% higher than the median skin knee temperatures. In synovitis and inflammatory disease, the intra-articular temperature was found to be around 35°C to 36°C [6]. At such temperatures, enzymatic activity (by cartilage-degrading cytokines such as IL-1 and IL-6, metalloproteinases, and other substances), may lead to cartilage damage. At intra-articular temperatures of, or slightly below 30°C, enzymatic cartilage damage in context of synovitis will be slight [27,28]. Oosterveld et al. found 30°C to be the threshold below which enzymatic activity and cartilage degradation were markedly reduced [29]. For cartilage damage to be caused, the temperature would need to be substantially below that threshold. Therefore he recommended to decrease intra-articular temperature in actively inflamed arthritic joints [6,30]. Increased intra-articular temperatures during sports activity in subjects with existing degenerative joint disease might lead to negative effects on the joint as found in synovitis. Therefore it raises the question if cooling during heavy demand of the knee joint should be recommended. However, temperatures around the physiological body temperature appear not to harm the articular cartilage in healthy knees. Cheng at al. evaluated the effects normal saline irrigation at different temperatures on the surface of articular cartilage in rats and found the most even surface without fibril exposure at 37°C.

Implantation of the probe with concomitant trauma and local anaesthesia to the knee appeared to decrease the ability of hopping in our study group and seemed to decrease joint position sense. Accordingly, less performance and increased risk for knee injury must be respected in the athlete with an acutely traumatized knee.

Cold or heat to a joint is of concern to have negative influence on muscle performance and increased risk of injury and therefore discussed controversial [15-21]. Hopkins et al. found the soleus motoneuron is facilitated following a 30-minute cooling period of the ankle and supported the application of ice to the ankle prior to activity and rehabilitation [17]. Sanya and Bello reported the quadriceps strength even to be improved after cooling and therefore recommended that application of cold on muscle should be employed while rehabilitating an individual with musculoskeletal pathology or deficit particularly while training for muscle endurance, strength and restoration of muscle function [20]. Other studies support these findings reporting no influence of local cooling on proprioceptive acuity in the quadriceps muscle and isometric force variability [31,32]. The length of the cooling period appears to have an important impact whether advantageous or disadvantageous muscle function is evident. 30 minutes of cooling (10°) showed improvement of maximal isometric grip strength immediately after the application of cold but decline by the end of treatment. The application of heat (40°) leads to decline of maximum strength during the first 22 minutes. However, this trend reversed itself with improvement of strength by the end [15]. Whereas cooling was reported to make knees stiffer and lessens the knee position sense [21], cryotherapy did not impair shoulder or ankle position sense [16,18]. Hopper et al. suggested that cryotherapy is not deleterious to joint position sense and assuming normal joint integrity patients may resume exercise without increased risk of injury [18]. Our data in a limited number of subjects revealed that different skin and intra-articular temperatures appear not to decrease joint function or laxity after skiing and jogging under different ambient temperature. Interestingly, the ability for hopping among our subjects seemed to be greater after skiing than after jogging. However, if this was related to the cooler intra-articular temperatures remains uncertain and needs to be proved in a larger number of subjects.

Limitations of the study must be considered and include the following. (1) The outside conditions at the skiing resort are not applicable as in a laboratory setting and are vulnerable to changes. (2) The skiing activity of the subject could not be standardised as the activity on the treadmill with preset speed. (3) Temperature measurements were done at a given time for all subjects at the skiing resort resulting in different time frames between activity and measurement. (4) The sample size of n = 6 was relatively small but sufficient to assess differences in intra-articular and knee skin temperatures when using non-parametric tests [33]. All data were tested for deviation from the normal distribution within the groups using Box-and-Whisker Plots. No extreme values were obtained indicating a normal distribution. Possible biases were reduced by using only male subjects of comparable age and BMI. The results of joint function and laxity, however, were of concern for insufficient power and therefore not eligible for statistical analysis.

## Conclusion

In conclusion, this study demonstrates different changes of intra-articular temperatures during sports in jogging and alpine skiing and suggests that changes are related to activity and ambient temperature. Further research is warranted to evaluate, if knees in diseased states are more vulnerable to negative effects of increased intra-articular temperatures in sports.

## Competing interests

In support of their research, one or more authors received grants or outside funding from Aircast, Inc., Summit, New Jersey, U.S.A. None of the authors received payments or other benefits or a commitment or agreement to provide such benefits from a commercial entity.

## Authors' contributions

CB was responsible for the manuscript preparation, participated in the design and conception of the study and the data analysis. JS participated in the conception of the study, supervising the protocol and data analysis. SF participated in the conception of the study, supervising the protocol, data analysis and critically reviewing earlier versions of the manuscript. GC participated in the initial conception of the research question, supervising the protocol and provided the infrastructure for the jogging setting. HHP was responsible for the initial conception of the research question, supervising the protocol, securing funding and critically reviewing earlier versions of the manuscript. All authors read, edited, and approved the final version of the manuscript.

## Pre-publication history

The pre-publication history for this paper can be accessed here:


